# Effects of short-term breathing exercises on respiratory recovery in patients with COVID-19: a quasi-experimental study

**DOI:** 10.1186/s13102-022-00451-z

**Published:** 2022-04-05

**Authors:** Manzur Kader, Md. Afzal Hossain, Vijayendar Reddy, Nirmala K. Panagodage Perera, Mamunur Rashid

**Affiliations:** 1 Institute of Environmental Medicine, Karolinska Institutet, Solnavägen 4, Torsplan floor 10, 113 65 Stockholm, Sweden; 2 Department of Physiotherapy, Zainul Haque Sikder Women’s Medical College and Hospital, Dhaka, Bangladesh; 3 Department of Neurosurgery, Zainul Haque Sikder Women’s Medical College and Hospital, Dhaka, Bangladesh; 4 Nuffield Department of Orthopaedics, Rheumatology, and Musculoskeletal Sciences, University of Oxford, Oxford, UK; 5 Unit of Physiotherapy, Department of Health, Medicine and Caring Sciences, Linköping University, Linköping, Sweden; 6 Department of Public Health and Sports Sciences, Faculty of Health and Occupational Studies, University of Gävle, Gävle, Sweden

**Keywords:** Breathing, Bangladesh, COVID, Chest PT, Oxygen saturation, Physiotherapy, Pulmonary exercise, Respiratory rehabilitation

## Abstract

**Background:**

Coronavirus disease 2019 (COVID-19) is a highly infectious respiratory tract disease. The most common clinical manifestation of severe COVID-19 is acute respiratory failure. Respiratory rehabilitation can be a crucial part of treatment, but data lack for patients with COVID-19. This study investigates the effects of short-term respiratory rehabilitation (i.e., breathing exercises) on respiratory recovery among non-ICU hospitalised patients with COVID-19.

**Methods:**

This was a quasi-experimental, pre-and post-test study. The study recruited 173 patients hospitalised with moderate to severe COVID-19. All the patients received standardised care for COVID-19, and 94 patients in the intervention group also received the intervention of breathing exercises, which included breathing control, followed by diaphragmatic breathing, deep breathing, or thoracic expansion exercise, and huffing (forced expiratory technique) and coughing. Data on the mean values of peripheral oxygen saturation (SpO_2_), need for oxygen therapy (litre/min), respiratory rate (breaths/minute), and heart rate (beats/minute) and were collected at baseline, 4 days, and 7 days after the baseline assessment. Analysis of variance on repeated measures was applied to compare the mean value of outcome measures of all the time points.

**Results:**

The mean (± SD) age of the intervention (69.6% men) and control group (62.1% men) were 50.1 (10.5) and 51.5 (10.4) years, respectively. At 4-day of follow-up, SpO2 (96.6% ± 1.9 vs. 90.7% ± 1.8, *P* < 0.001), need for oxygen therapy (0.8 ± 2.6 vs. 2.3 ± 2.9, *P* < 0.001), respiratory rate (20.5 ± 2.3 vs. 22.3 ± 2.5, *P* < 0.001), and heart rate (81.2 ± 9.5 vs. 89.2 ± 8.9, *P* < 0.001) improved in the intervention group compared to the control group. At 7-day follow-up, differences remained significant concerning the oxygen saturation and the need for oxygen therapy (*P* < 0.001) between the groups.

**Conclusions:**

Our results indicate that breathing exercise, even for a short period, effectively improves specific respiratory parameters in moderate to severe COVID-19 patients. As a non-invasive and cost-effective respiratory rehabilitation intervention, breathing exercise can be a valuable tool for a health care system overwhelmed by the COVID-19 pandemic. These results should be considered preliminary until they are replicated in larger samples in different settings.

## Background

Coronavirus disease 2019 (COVID-19) is a highly infectious respiratory tract disease caused by severe acute respiratory syndrome coronavirus 2 (SARS-CoV-2) [1, 2].

According to WHO COVID-19 disease severity classification for adults, the moderate disease is defined as clinical signs of pneumonia (fever, cough, dyspnoea, fast breathing) but no signs of severe pneumonia, including peripheral oxygen saturation (SpO2) ≥ 90% on room air. Adults with severe disease are those with clinical signs of pneumonia (fever, cough, dyspnoea) plus one of the following: respiratory rate > 30 breaths/min; severe respiratory distress; or SpO2 < 90% on room air [1]. According to this classification, those who become symptomatic with COVID-19, most people develop only mild (40%) or moderate (40%) disease, approximately 15% develop a severe disease that requires oxygen support, and 5% have a critical disease with complications such as respiratory failure, acute respiratory distress syndrome (ARDS), septic shock, thromboembolism, and/or multi-organ failure [1].

Evidence shows that the most common clinical presentation of severe COVID-19 is viral pneumonia featuring fever, cough, dyspnoea, hypoxemia, and bilateral infiltrates on chest radiographs [2–4]. Peripheral oxygen saturation of 92% or lower is one defining feature of moderate to severe disease in acute COVID-19 requiring urgent referral to hospital [5]. As mentioned before, severe respiratory symptoms can cause respiratory failure (ARDS), which can lead to death unless promptly managed using ventilation at ICU [2, 3, 6]. Mortality associated with COVID-19 ranges from 16 to 78% among all infected persons [2, 7, 8].

Respiratory rehabilitation is crucial for the recovery of patients with viral pneumonia from COVID-19 during the acute and rehabilitation phases [6, 9]. Respiratory rehabilitation includes breathing exercises and respiratory muscle training using diaphragmatic breathing, pursed-lip breathing, relaxation, and body position exercises [10–13]. Respiratory rehabilitation improves the physical and psychological symptoms of lung diseases such as chronic obstructive pulmonary disease (COPD) [11]. It may improve oxygen exchange, prevents the lungs from collapsing, reduce psychological stress and the need for artificial ventilation [10–12]. A recent scoping review, including 40 recent publications, mainly guidelines, recommendations, perspectives, opinion letters, correspondence, and position papers, has highlighted pulmonary rehabilitation (or respiratory rehabilitation) in COVID-19. The review found that respiratory rehabilitation appears to be useful in COVID-19 survivors, and respiratory rehabilitation starting already in a hospital may lead to improved overall respiratory function and reduced hospital stay. However, the authors concluded a paucity of high-quality research on this topic [13]. A recent randomised controlled trial of six-week respiratory rehabilitation reported significant improvement to certain respiratory functions (e.g., forced expiratory volume in 1 s (FEV1), forced vital capacity (FVC)), QoL, and anxiety in elderly patients (≥ 65 years) with COVID-19 without COPD [14]. At the time of writing, there is limited evidence about the effects of respiratory rehabilitation in the acute stage of COVID-19 treatment and cardio-respiratory recovery in patients with COVID-19 [15–18]. This is because the respiratory problems experienced by patients with COVID-19 significantly differ from other respiratory conditions (e.g., dry cough is common [4, 19]) and COVID-19 patients' rapid deterioration to acute respiratory failure [2–4]. Also, the availability of health resources to treat COVID-19 patients in low-resource settings may be limited. As a cost-effective intervention, the impact of short-term respiratory rehabilitation on respiratory parameters in COVID-19 patients in resource-poor settings is not previously established. Therefore, this study aims to examine the effects of short-term breathing exercises on respiratory recovery (i.e., oxygen saturation, respiratory rate (breaths/minute), and heart rate (beats/minute), and oxygen therapy (litre/min)) among hospital-admitted patients with COVID-19. We hypothesised that the breathing exercise intervention would result in significant improvements in the outcome measure, compared to the control group.

## Methods

### Study design and population

We used a quasi-experimental design with pre-and post-tests [20] in non-ICU hospitalised patients with a laboratory-confirmed COVID-19 positive with reverse transcription-polymerase chain reaction between 3 May 2020 and 27 January 2021.

### Inclusion and exclusion criteria

All consecutive spontaneously breathing non-ICU patients, without any respiratory support (invasive or non-invasive), but required oxygen supplementation, aged between 18 and 70 years, were screened for enrolment. The study included only the patients with moderate to severe COVID-19-related respiratory symptoms because respiratory rehabilitation was recommended only for these patients according to previous literature [15–18]. The moderate to severe conditions of COVID-19 was based on the baseline peripheral oxygen saturation (SpO2) lower than 93%. A total of 237 patients met the general inclusion criteria: age 18–70 years, and having oxygen saturation < 93% and needing any form of oxygen supplementation (< 10 L/min) [5, 21]. The inclusion eligibility in the study was screened by nurses, physicians, or physiotherapists. Of 237 patients who were primarily identified as eligible to the study, 64 patients were excluded before the allocation to the intervention and the control group based on the following recommendations for respiratory rehabilitation intervention to the non-ICU patients [9, 17, 18]:

- Declined to participate (*n* = 11),
- Had SpO2 < 80% or needed oxygen > 10 L/min at baseline, or admitted to ICU (*n* = 30),
- Recent stroke (*n* = 1), pulmonary emphysema (*n* = 2), deceased (*n* = 3), and
- Other reasons (high fever, diarrhoea, hypotension, *n* = 17).

**Fig. 1:**
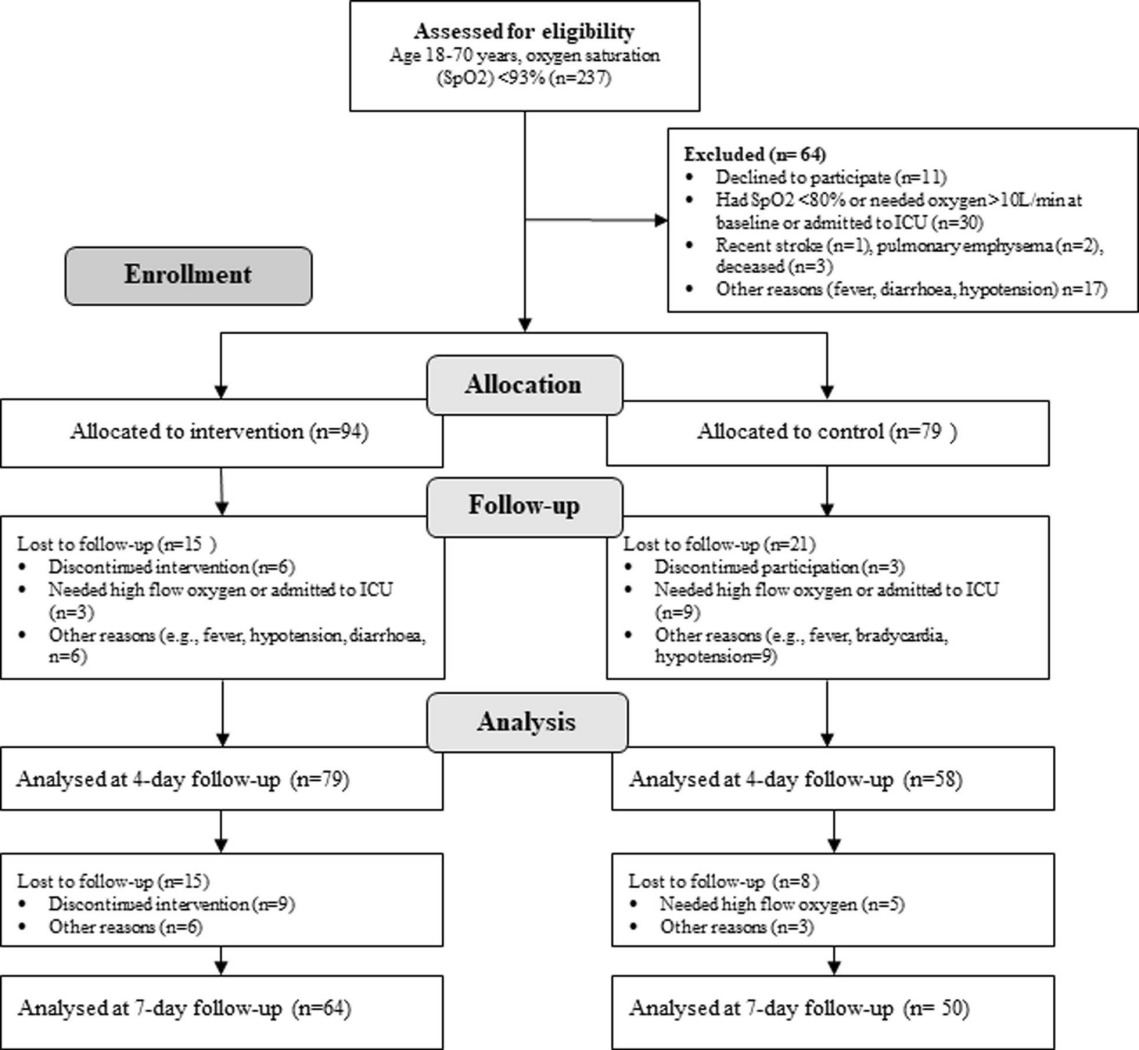
Flow diagram of patients through phases of a 2-group parallel quasi-experimental design. *ICU* intensive care unit

Consequently, 94 patients were allocated to the intervention, and 79 patients were allocated to the control group. See details of the recruitment process of patients in Fig. 1. A total of 79 persons in the intervention and 58 persons in the control group were analysed at 4-day of follow-up, and 64 persons in the intervention and 50 persons in the control group were analysed at 7-day of follow-up (Fig. 1).

### Group allocation

The study participants (intervention and control group) were recruited from tertiary hospitals in Bangladesh. These hospitals had government authorisation to provide standardised care to COVID‐19 patients according to the National Guidelines on Clinical Management of COVID-19 [22]. For example, all patients received symptomatic treatment, oxygenation support (if SpO_2_ < 93%), antiviral (e.g., Remdesivir), high flow oxygen, and mechanical ventilation for acute respiratory distress. In addition to the standardised care [22], few hospitals provided respiratory rehabilitation to patients admitted with severe COVID‐19, while others who treated similar patients were chosen to stick with the usual protocol of the standardised care. We selected a group of patients to study the respiratory recovery of the patients treated with standardised care together with respiratory rehabilitation (intervention group) versus those receiving the standard treatment only were allocated to the control group.

Patients who attended one tertiary hospital with respiratory rehabilitation between 1 June to 19 January 2021 and met the above eligibility criteria were recruited to the intervention group. The Control group was recruited from three other tertiary hospitals between 3 May 2020 and 27 January 2021. See details in Fig. 1.

We selected all these hospitals based on the criteria that they have equally trained qualified health care workers (e.g., nurses, physicians, or physiotherapists) for patients' assessment and data collection. Both groups (intervention and control) received a standard protocol for data collection, such as the same types of equipment, the same time of the day, and ensure comparability among the measures in the respective hospital. The elected hospital for intervention consisted of a well-trained physiotherapists' team in the breathing exercise intervention.

### Intervention

We provided respiratory rehabilitation according to the recently developed guidelines for patients with COVID-19 in the acute hospital setting [9, 15, 17, 23]. We started interventions for the patients within 6 h of the baseline assessment. At the beginning of the intervention, the patients were advised to avoid a supine position and maintain a prone position for at least 6–8 h (but not continuous) in a day. During the rest of the time, a lateral side-lying was maintained according to the current recommendation [23]. The intervention group received a range of breathing exercises in suitable positions [10, 12]. A physiotherapist supervised each breathing exercise session, and one cycle of breathing exercise included: (1) breathing control, followed by (2) diaphragmatic breathing, (3) deep breathing or thoracic expansion exercise, and (4) huffing (forced expiratory technique) and coughing. For breathing control, patients were instructed to breathe in and out gently through the nose. If they were unable to do that, they were encouraged to breathe through the mouth instead. Patients were given counseling for trying to let any tension/distress in the body with each breath out, and at the same time keep the shoulders relaxed and gradually try to make the breaths slower. Usually, six breathes at each session were continued until the patients felt ready to progress to the other stages. For diaphragmatic breathing, patients performed ten diaphragmatic breathing sitting in the relaxed position. In this position, they made their knee bent, neck and shoulder relaxed, placing one hand on the upper chest wall and another hand just below the rib cage; afterward, take a breath in through the nose for 3 s and out through the mouth for 3 s, and take a normal breath between two consecutive sessions. For the deep breathing exercise, patients were instructed to take a slow, long, and deep breath through the nose, hold air for 2–3 s before breathing out, and then to breathe out gently. Then the patients relaxed like a sigh without forcing the air out, in a supine lying position with the knees semi flexed with pillow support. For huffing and coughing, two sets of 2–3 active huffing and coughing were done after deep breathing exercise in the same position, with a one–minute rest between the two sets. Moreover, we added an incentive Spirometer for the patients whose respiratory rate was below 25/min. It was around 10 min long and given four times daily.

The patients were in a prone position for at least 30 min after completing each breathing exercise session. Each session was around 15 min long, applied between 8.00 am and 10.00 pm, a minimum of 1.5 h after a meal. Each patient participated actively under the supervision of a physiotherapist. Around seven sessions were given every day. Among all sessions, at least three sessions were delivered by direct supervision of the Physiotherapist. The rest of the sessions were delivered with the assistance of a trained nurse or nursing assistant and were recorded in an activity log. The Physiotherapist gave verbal and written instruction guidelines. During the first 4-day follow-up, the intervention group received a constant breathing exercise session according to the guideline. It should be noted that the intervention between 5 and 7 days was tailored to the individual patient's needs (i.e., frequency, intensity, and timing of the interventions). It means that from 5-day to 7-day of follow-up, the frequency, intensity, duration, and number of intervention sessions might have been reduced according to the individual's daily progress report in outcome measures.

All the physiotherapists paid particular attention during the interventions that may expose them to a higher risk of contamination due to the dispersion of droplets in the air [24]. For example:

- Wearing personal protective equipment such as an N95 mask, fluid-resistant long-sleeve gown, goggles/face shield, and gloves,
- Cough etiquette and hygiene such as patient to turning head away during cough and expectoration and when possible, Physiotherapist positioned themselves ≥ 2 m from the patient and out of the "blast zone" or line of cough,
- The Physiotherapist avoiding aerosol-generating procedures. They implemented any non-invasive ventilatory support in particular with open masks or other open systems, first obtaining agreement with senior Physicians.

### Data collection

Patients' baseline data were collected from hospital-record. These included sociodemographic data (e.g., age, sex, education), haematological/biochemical data (e.g., lymphocyte, serum D-Dimer, ferritin), and presence of any major coexisting illnesses and/or diseases (e.g., COPD, diabetes). The baseline assessment was done within 24 h of hospital admission. However, the baseline assessment of SpO2, respiratory rate, and heart rate were taken upon arrival to the ward or preadmission, usually within 30 min, to evaluate whether a patient needs oxygen supplementation or hospitalisation.

### Outcomes measure

As outcomes in the study, we collected respiratory clinical parameters: SpO_2_, need for oxygen therapy, respiratory rate, and heart rate at baseline (before the treatment), and followed them up at two-time points (4 days and 7 days). These outcomes were monitored and recorded several times a day, especially oxygen saturation and heart rate every hour. For the present study, we measured the outcomes when a patient was in a complete resting position at around 10.00 am, at baseline, and 4 days and 7 days after the baseline assessment for the intervention and the control group. However, the measurements were taken at least 30 min after any breathing exercise session for the intervention group.

### Procedure

The SpO_2)_ was measured using the adult finger pulse oximeter PM100C (New Tech^®^, EUA), positioned on the hand's fifth finger with the patient in the upright (sitting) position and resting. The SpO_2_ indicates the percentage of arterial haemoglobin saturated with oxygen and is a vital sign [25].

The need for oxygen therapy was recorded as litres/minute. A face mask was used to deliver oxygen flow up to 5 L/min, a reservoir mask up to 10 L/min of oxygen[15].

Respiratory rate was recorded by counting the number of breaths/minutes is an early indicator of hypoxia, hypercapnia, and metabolic and respiratory acidosis [6, 26].

Heart rate (beats/minute) was assessed by measuring the radial pulse. The regularity of heart rhythm indicates the strength of heart contraction and sufficiency of cardiac output.

### Statistical analysis

Descriptive statistics were computed for all the variables. Continuous variables were expressed by mean and standard deviation and tested using an independent *t* test between groups. Categorical variables were described as frequency and percentage and tested using the Chi-square test between groups. The sample of each group was large enough, and comparisons were not affected by the shape of the error distribution and no transformation was applied [27]. A two-way analysis of variance on repeated measures (with Bonferroni post hoc adjustment), and paired t-test on each intervention or control group were applied to compare the outcomes of each respiratory parameter at baseline 4 days, and 7 days after the baseline assessment. The number of participants included in the study was determined by Power analysis done in G*Power (version 3.1.9.4.). A priori power analysis for a repeated-measures analysis of variance with two repeated-measures showed that a total of 108 participants would require to get a statistical power (1 − *β* err prob) of 80%.

All reported *P* values are based on two-sided tests, with a *P* value of less than 0.05 considered as significant. All the data were analysed using the IBM SPSS Statistics for Windows, version 26 (IBM Corporation, Released 2019, Armonk, 137 New York, United States).

## Results

### Participant characteristics

The intervention group included 79 patients (69.6% men, mean age 50.1 ± 10.5 years), and the control group included 58 patients (62.1% men, mean age 51.5 ± 10.4 years). The sociodemographic- and hematological data, and presence of comorbidity did not differ statistically between the intervention and the control groups (*P* > 0.05), except for the total White Blood Cell (WBC) count that was found higher in the intervention group, compared to the control group (*P* = 0.04). (Table 1).

**Table 1:** Baseline characteristics of participants with COVID-19 patients: intervention versus control group (N = 137)

Variables	Intervention group *n* = 79	Control group *n* = 58	*P* value^a^
*Demographics*			
Age, mean (± SD)	50.1 (10.5)	51.5 (10.4)	0.43
Sex (men), *n* (%)	55 (69.6)	36 (62.1)	0.36
BMI, kg/m^2^, mean (± SD)	25.7 (4.6) *missing (n* = *5)*	27.1 (5.4) *missing (n* = *9)*	0.17
Employment status (employed)	61 (77.8)	47 (81) *missing (n* = *3)*	0.65
Education			0.63
Secondary or above, *n* (%)	69 (87.3)	49 (84.5)	
Primary or no formal education, *n* (%)	10 (12.7)	9 (15.5)	
Current smoker (yes), *n* (%)	15 (19)	10 (17.2)	0.86
*Haematological/biochemical data*			
Hemoglobin, gm/dL, mean (± SD)	12.84 (2.1)	12.67 (1.8)	0.62
Total WBC, mcL, mean (± SD)	8260.8 (4073.1)	9818.9 (4562.2)	0.04
Neutrophil, mcL, mean (± SD)	6335.1 (3987.1) *missing (n* = *1)*	7533.3 (4287.9)	0.10
Lymphocytes, mcL, mean (± SD)	1549.4 (735.6) *missing (n* = *1)*	1759.9 (1191.3)	0.21
SGPT, units /L, mean (± SD)	50.45 (27.6) *missing (n* = *29)*	39.03 (27.5) *missing (n* = *19)*	0.06
Serum D-Dimer, μg/ml,mean (± SD)	0.41 (0.5) *missing (n* = *9)*	0.47 (0.4) *missing (n* = *20)*	0.48
Serum ferritin, ng/ml, mean (± SD)	293.6 (294.7) *missing (n* = *9)*	290.0 (367.8) *missing (n* = *20)*	0.95
C-reactive protein, mg/L, mean (± SD)	16.9 (27.9) *missing (n* = *9)*	19.7 (27.9) *missing (n* = *20)*	0.58
*Disease related data/comorbidities*			
Need of oxygen supplementation			0.15
1–4 L/min, *n* (%)	62 (78.5)	51 (87.9)	
5–10 L/min, *n* (%)	17 (21.5)	7 (12.1)	
Productive cough (yes), *n* (%)	21(26.6)	18 (31)	0.57
Able to clear secretions independently (yes), *n* (%)	18 (22.8)	18 (31)	0.32
COPD or other respiratory diseases, (yes), *n* (%)	24 (30.4)	18 (31)	0.54
*Other major comorbidities*			
Type 2 diabetes, *n* (%)	49 (62)	40 (69)	0.47
Hypertension, *n* (%)	51 (64.6)	38 (65.5)	0.52
Ischemic heart disease, *n* (%)	5 (6.3)	6 (10.3)	0.29
Kidney diseases, *n* (%)	6 (8.6)	4 (6.9)	0.49
Liver diseases, *n* (%)	2 (3.0)	2 (3.4)	0.63
Malignant tumor, *n* (%)	3 (4.5)	0 (0)	0.15

COVID-19, Coronavirus 2019; WBC, White blood cells; SGPT, Serum Glutamic-Pyruvic Transaminase (Liver Function Tests); COPD, chronic obstructive pulmonary disease. ^a^Differences were assessed with independent *t* test for continuous variables, and Pearson’s Chi-square tests for categorical variables

### Changes in respiratory parameters

At the baseline, no significant differences were found (*P* > 0.05) in the mean values of SpO2, the need for oxygen, respiratory rate, and heart rate between the intervention and the control group (Table 2). After 4 days of breathing exercises, the mean SpO2 (96.6 ± 1.9 vs. 90.7 ± 1.8), *P* < 0.001), the need for oxygen (0.81 ± 2.6 vs. 2.3 ± 2.9 L/min, *P* < 0.001), respiratory rate (20.5 ± 2.3 vs. 22.3 ± 2.5 breaths/min, *P* < 0.001), and heart rate (81.2 ± 9.5 vs. 89.2 ± 8.9 beats/min P < 0.001) improved in the intervention group compared to the control group (Table 2).

**Table 2:** Comparison of oxygen saturation, the need for oxygen, respiratory rate, and heart rate between the intervention and control groups at baseline (pre) and 4-day of follow-up (post)

	Intervention group, (*n* = 79)			Control group, (*n* = 58)			Between group comparison (Intervention-control)	
Measures	Pre	Post	Within group *P* value	Pre	Post	Within group *P* value	Pre *P* value	Post *P* value
Oxygen saturation (SpO2) mean (± SD)	87.9 (3.7)	96.6 (1.9)	< 0.001	88.0 (2.4)	90.7 (1.8)	< 0.001	0.75	< 0.001
Need of oxygen, (litre/min) mean (± SD)	2.9 (2.8)	0.81 (2.6)	< 0.001	2.1 (2.6)	2.3 (2.9)	0.32	0.06	< 0.001
Respiratory rate (breaths/min), mean (± SD)	26.8 (5.2)	20.5 (2.3)	< 0.001	25.4 (3.9)	22.3 (2.5)	< 0.001	0.08	< 0.001
*Missing*	*n* = *10*	*n* = *12*		*n* = *7*	*n* = *9*			
Heart rate (beats/min) mean (± SD)	93.3 (12.7)	81.2 (9.5)	< 0.001	95.6 (9.3)	89.2 (8.9)	< 0.001	0.26	< 0.001
*Missing*	*n* = *1*	*n* = *0*		*n* = *1*	*n* = *2*			

**Fig. 2:**
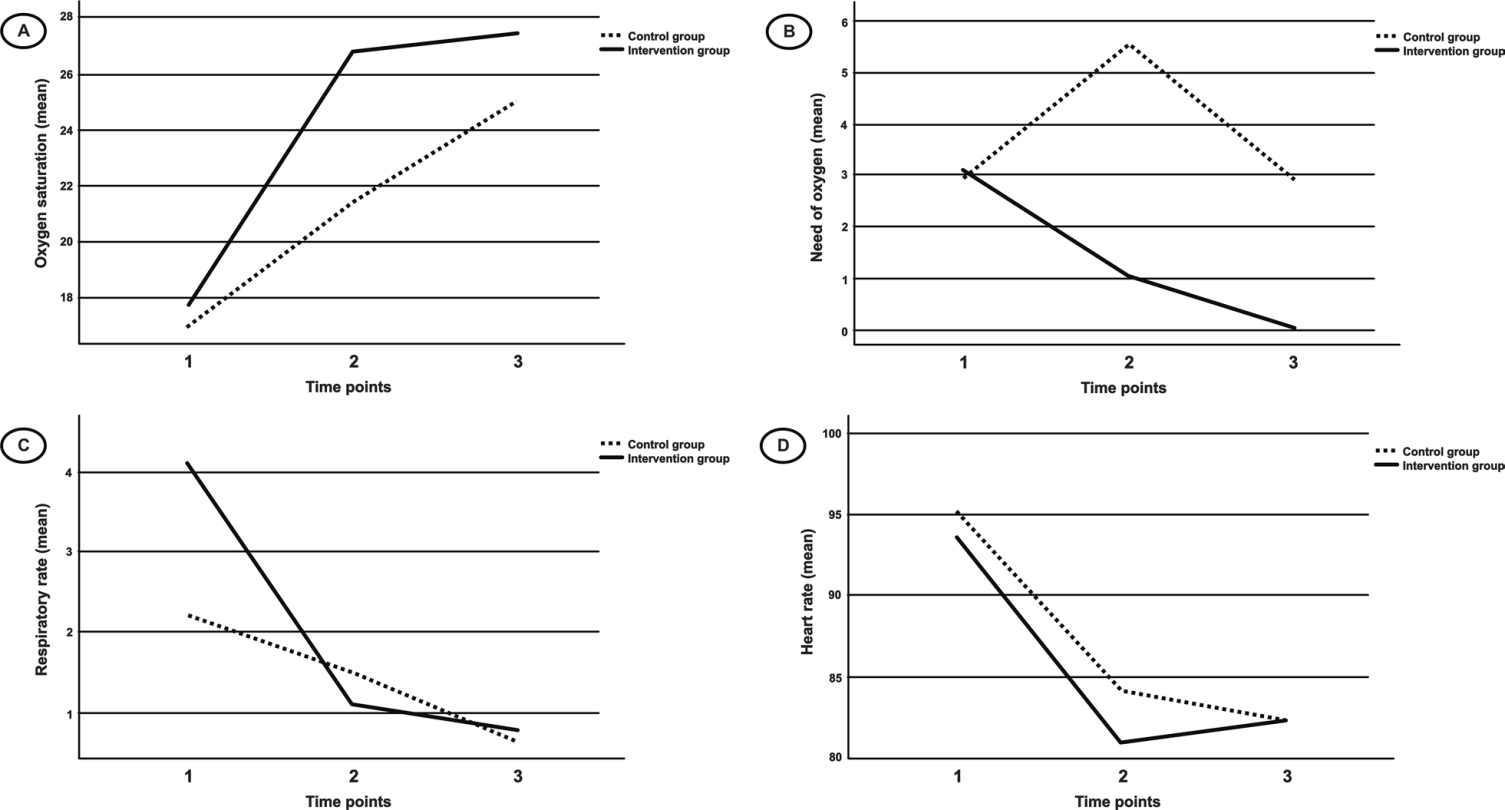
**A** Oxygen saturation (SpO2) over three time points (i.e., 1 = baseline; 2 = 4-day follow-up; 3 = 7-day follow-up) between groups (i.e., intervention and control group) (*P* < 0.001 at the time point 2 and 3); **B** Need of oxygen (liter/minute) over three time points between groups (*P* < 0.001 at the time point 2 and 3); **C** Respiratory rate (breaths/minute) over three time points between groups (*P* < 0.001 at the time point 2, and *P* = 0.09 at the time point 3); **D** Heart rate (beats/minute) over three time points between groups (*P* < 0.001 at the time point 2, and *P* = 0.47 at the time point 3)

Figure 2 represents the differences in mean values of respiratory parameters between the groups at 4-day and 7-day follow-up. After 7 days of follow-up from the baseline, a significant difference in SpO_2_ and the need for oxygen therapy (*P* < 0.001) were observed between the groups (intervention and control). However, the difference in respiratory rate (*P* = 0.09) and heart rate (*P* = 0.47) did not appear significant between the groups (Fig. 2).

## Discussion

One of the key findings of the present study was that SpO_2_, respiratory rate, and heart rate improved in patients with COVID-19 who received breathing exercise while reducing their need for oxygen therapy. To the best of our knowledge, this is the first study to investigate the short-term effect of breathing exercises on respiratory recovery in patients with COVID-19, using a quasi-experimental study design. Only one previous study among elderly patients (≥ 65 years) with COVID-19 without COPD using a randomised controlled trial of six-week respiratory rehabilitation found improvement in SpO_2_ [14], and our findings concur.

The results supported our original hypothesis that respiratory parameters would improve in both intervention and control groups, with the intervention group demonstrating more significant improvements compared to the control group. The physiological rationale behind the progress might be that breathing exercise improves respiratory muscle function, ribcage flexibility, gas exchange, and may decrease blood pressure, respiratory rate, and stress [11, 12, 28, 29]; consequently, helping patients with COVID-19 to manage their respiratory symptoms. A systematic review of randomized controlled trials and two quasi-experimental studies in adults suggests that diaphragmatic breathing may decrease blood pressure, respiratory rate, and psychological stress [12]. Inspiration through the nose should be encouraged to facilitate the recruitment of the diaphragm and improve humidification [29]. A forced expiratory technique like huffing and coughing increases the linear velocity of the expiratory airflow and propels secretions, which help in airways clearance [28]. Suitable positioning may be used to enhance ventilation, perfusion, oxygenation, and mobilization of tracheobronchial secretions via gravitational effects [23, 30]. We encouraged the patients to be in a prone position several hours a day. Based on the current recommendation, it has been theorized that adopting the prone position for conscious, non-intubated patients with COVID-19 helps improve oxygenation, reduce the need for invasive ventilation and potentially decrease mortality [23]. However, the patients can be in all suitable positions including prone, side-lying, upright, supine, and guided by the location of consolidations seen on imaging or found on examination [31].

Both scientific and anecdotal reports have highlighted the importance of breathing exercises for maintaining respiratory function [15–18]. In a randomised controlled trial, elderly patients (aged ≥ 65) with COVID-19 without COPD improved their SpO_2_ following respiratory rehabilitation[14], and our findings concur. Given the high respiratory impairment burden following the acute phase of COVID-19, patients should be referred early to a respiratory rehabilitation programme, particularly those admitted to a hospital.

Abdominal chest imaging, and severe impairment to pulmonary diffusion capacities was reported in COVID-19 patients recovered from severe illness [32]. Thus, how respiratory rehabilitation in the acute phase impacts long-term recovery should be explored in future studies. Further, severe lung complications from COVID-19 may have persisting limitations to respiratory function and gas exchange; this group of patients, therefore, should be the primary target population for the intervention of long-term recovery. Roles of respiratory rehabilitation programs via outpatients' services and via primary care should be further explored.

It should be noted that the COVID-19 patients included in the intervention group in our study were sufficiently stable during the study period, and only three patients needed to admit to the ICU after the intervention started. It has previously been recommended that breathing exercises be stopped for chest pain, palpations, and dizziness and be stopped if SpO2 does not recover even with rest and oxygen supplementation [33]. Moreover, appropriate infection control strategies must be employed to prevent droplet contamination by coughing, sneezing, and close contact with a COVID-19 patient's during treatment. Typically, a non-productive cough is associated with COVID-19; productive coughing may appear at a later stage [19]. Therefore, as a precaution, airways should be regularly cleared to remove bronchial secretions. Respiratory rehabilitation should be considered when there are no signs of progressive deterioration and patients are hospitalised, as recommendations by Chinese, the Netherlands, Italian, and UK rehabilitation professionals [6, 10, 15, 17, 33].

### Clinical implications

In patients with COVID-19, low blood oxygen levels are associated with rapid deterioration to acute respiratory distress or failure, leading to death unless it is managed immediately [2–4]. We found that respiratory rehabilitation during the acute phase of care improves SpO_2_, respiratory rate, and heart rate in patients hospitalised with COVID-19. Our study guides the delivery of quality respiratory care to patients with COVID-19. Rapidly increasing demand for healthcare, including intensive care, has placed unprecedented strain on the health system across the globe. Respiratory rehabilitation is non-invasive, safe, and easy to implement, and cost-effective. Health care systems are overwhelmed in many countries; respiratory rehabilitation thus provides some respite.

An individual approach to respiratory rehabilitation led by a multidisciplinary team (e.g., physician, Physiotherapist, occupational therapist, and nurses) can increase positive outcomes [6, 15, 19]. Furthermore, determining health care resources such as bed, staff, equipment, and therapeutic is a key priority for many countries as the COVID-19 escalate. Given the risk of infection, physiotherapists and other health care staff administering respiratory rehabilitation need to take appropriate steps such as wearing personal protective equipment to protect themselves from droplet contamination by coughing and sneezing during breathing exercises.

### Strength and limitation

The key strengths of this study are methodological rigour, using quasi-experimental design when it was not logistically feasible or ethical to conduct a randomised controlled trial. Like the randomised controlled trial, the quasi-experimental design can establish causal associations between an invention and an outcome [34]. But because participants are not randomly assigned, making it likely that there are other differences between conditions. Another strength is the representative sample size from both sexes and sociodemographic background (e.g., educational level, employment), with broad age groups (18–70 years). We included a large amount of hematological and disease-related baseline data to ensure comparability among the intervention and the control group. Further patients with COVID-19 were not excluded based on pre-specified comorbidities (e.g., COPD). Thus, our findings apply to similar populations. Study participants were recruited from four tertiary hospitals from Bangladesh; the intervention group from one hospital, and the control group from the other three hospitals. Therefore, resource availability might differ between hospitals. However, the standardised care was provided to all patients according to the National Guidelines on Clinical Management of COVID-19 [22], and we followed a standard protocol for data collection (e.g., same instrument, same time of the day, same order) to ensure comparability among the measures collected in different hospitals. Only the short-term effects of 4-day and 7-day of intervention were evaluated, which is a limitation of our data. Another limitation of our study is that no specific information about the number of interventions was collected between the 5-day and 7-day of follow-up, as the intervention was varied or reduced based on patients' needs during this follow-up period.

## Conclusions

Rapidly increasing demand for healthcare, including intensive care, has placed unprecedented strain on the health system across the globe. Breathing exercise as a part of respiratory rehabilitation improved respiratory parameters in patients hospitalised with COVID-19. These results should be considered preliminary until they are replicated in larger samples in various settings. Further studies are warranted to determine the long-term effect of breathing exercises on the overall respiratory functions in patients with COVID-19. However, respiratory rehabilitation is non-invasive, safe, easy to implement, and cost-effective. Health care systems are overwhelmed in many countries; respiratory rehabilitation thus provides some respite. However, given the highly contagious nature of the SARS-CoV-2, a robust respiratory rehabilitation plan must be in place to make optimal use of a limited rehabilitation workforce and reduce risk to health professionals.

## Data Availability

The datasets for the current study are available from the corresponding authors upon reasonable request.

## Abbreviations

COVID-19: Coronavirus disease 2019
SARS-CoV-2: Severe acute respiratory syndrome coronavirus 2
ICU: Intensive Care Unit
ARDS: Acute respiratory distress syndrome
COPD: Chronic obstructive pulmonary disease
QoL: Quality of life
FEV1: Forced expiratory volume in 1 s
FVC: Forced vital capacity
SpO2: Peripheral oxygen saturation
